# Effects of environmental distractors on nurse emergency triage accuracy: a pilot study protocol

**DOI:** 10.1186/s40814-020-00717-8

**Published:** 2020-11-07

**Authors:** Philippe Delmas, Assunta Fiorentino, Matteo Antonini, Séverine Vuilleumier, Guy Stotzer, Aurélien Kollbrunner, Dominique Jaccard, Jarle Hulaas, Olivier Rutschmann, Josette Simon, Olivier Hugli, Charlotte Gilart de Keranflec’h, Jérome Pasquier

**Affiliations:** 1 La Source School of Nursing, University of Applied Sciences and Arts Western Switzerland (HES-SO), Lausanne, Switzerland; 2 School of Management and Engineering Vaud, Yverdon-les-Bains, Switzerland; 3 Emergency Department, Geneva University Hospital, Geneva, Switzerland; 4 Emergency Department, Lausanne University Hospital, Lausanne, Switzerland; 5 School of Health Sciences (HESAV), University of Applied Sciences and Arts Western Switzerland (HES-SO), Lausanne, Switzerland; 6 Institute of Social and Preventive Medicine, Lausanne University Hospital, Lausanne, Switzerland

**Keywords:** Serious game, Triage accuracy, Emergency department, Distractors, Patient safety

## Abstract

**Background:**

The clinical decisions of emergency department triage nurses need to be of the highest accuracy. However, studies have found repeatedly that these nurses over- or underestimate the severity of patient health conditions. This has major consequences for patient safety and patient flow management. Workplace distractors such as noise and task interruptions have been pointed to as factors that might explain this inaccuracy. The use of a serious game reproducing the work environment during triage affords the opportunity to explore the impact of these distractors on nurse emergency triage accuracy, in a safe setting.

**Methods/design:**

A pilot study with a factorial design will be carried out to test the acceptability and feasibility of a serious game developed specifically to simulate the triage process in emergency departments and to explore the primary effects of distractors on nurse emergency triage accuracy. Eighty emergency nurses will be randomized into four groups: three groups exposed to different distractors (A, noise; B, task interruptions; C, noise and task interruptions) and one control group. All nurses will have to complete 20 clinical vignettes within 2 h. For each vignette, a gold standard assessment will be determined by experts. Pre-tests will be conducted with clinicians and certified emergency nurses to evaluate the appeal of the serious game.

**Discussion:**

Study results will inform the design of large-scale investigations and will help identify teaching, training, and research areas that require further development.

## Background

Patient safety is a key concern amid continuous efforts to improve the effectiveness and efficiency of health systems [1]. Defined as the “reduction of risk of unnecessary harm associated with health care to an acceptable minimum” [2] (p. 19), patient safety refers to the presence or absence of adverse events and/or medical errors and serves to compare the performance of health facilities worldwide [3]. Patient safety is strongly related to different nurse activities, particularly those where clinical decisions must be made [4, 5]. Clinical decision-making has been defined as “a contextual, continuous, and evolving process, where data are gathered, interpreted, and evaluated in order to select an evidence-based choice of action” [6] (p. 401).

Decision-making is a frequently performed process in emergency departments, especially in the course of triage, when nurses sort patients by care priority based on a process that aims for the best allocation of human and physical resources. It remains a human-driven process and as such is prone to error. Errors in clinical decisions can have serious consequences for patient safety [7, 8]. Underestimating the severity of a patient’s condition can lead to delayed emergency care and medical treatment that decreases the quality of the intervention [9]. Overestimating a patient’s condition results in inadequate resource allocation, which leads to overcrowding and work overloads in specific units [10]. With this in mind, nurses must arrive at highly reliable decisions and limit the occurrence of errors. What is more, nurses who perform triage must not only reach accurate decisions, they must also do so rapidly [11–13]. Indeed, decisions must be made within a very short time lapse (< 5 min) based on limited information and with limited access to peer clinical supervision [11, 14, 15].

To support nurses during the ED triage process and reduce errors, various scales have been developed since the early 1990s. Today, most of the existing triage scales cover four or five emergency levels [16–18]. Following these international standards, the Swiss Society of Emergency and Rescue Medicine currently recommended using the Swiss Emergency Triage Scale or SETS® [19] for ED patient triage. The SETS® has been psychometrically validated [10, 20] and is widely used in Switzerland, France, and Belgium. It spans four emergency levels from 1 to 4: acute, urgent, semi-urgent, and non-urgent. The introduction of triage scales has standardized nurse decisions and has facilitated the study of triage quality. Triage quality is most commonly assessed according to one indicator: accuracy. The accuracy of nurse triage decisions is measured by the degree of agreement between the emergency level assigned and a gold standard set by a panel of experts [21]. When nurses assign a level higher than the gold standard, it is referred to as overtriage [22, 23]. When the assigned level is lower, it is referred to as undertriage [9, 24].

In their systematic review, Farrohknia et al. [25] found the level of accuracy of nurse emergency level assignments to be medium to low. There has been little research, however, into the reasons for this relatively poor performance. The few studies that have investigated the issue have done so from the angle of nurse individual factors and contextual factors [26–28]. The individual factors considered include characteristics specific to nurses such as personality (flexibility, decision-making autonomy), cognitive processes (critical thinking, prompt decision-making), behavioral processes (working under pressure, being organized), and nurse experience (confidence in one’s decision-making) [28–30]. Where contextual factors are concerned, researchers have focused on the numerous distractors present in the environment [31–34], specifically frequent task interruptions, noise, and variable workloads. Any of these can result in the delayed performance of care activities, information loss, and a drop in concentration that alters the decision-making process, particularly when performing complex activities.

On a recurring basis, researchers who have examined the clinical decision-making of emergency nurses have generally used one of two approaches: retrospective reviews or nurse-assessed written clinical vignettes. Both methodologies have their shortcomings. For example, written vignettes present limited cues to make accurate decisions and retrospective reviews must cope with missing data [29, 35]. To reduce such biases, some authors [36] have underscored the importance of reproducing real-world conditions as faithfully as possible and of placing nurses in these circumstances. In this regard, the use of serious games (SG) simulating nursing tasks and the ED triage environment provides researchers with a unique opportunity to immerse nurses in such situations [35, 36] and investigate factors affecting triage, in a safe environment.

Nowadays, SG appear to be a solution for exploring clinical decision-making in the context of simulation [37, 38]. SG are defined as games whose primary purpose is neither fun nor entertainment. These are frequently used in the context of professional development and training, education, and scientific research [39, 40] to develop competencies in fields where poor decisions are associated with a high adverse-event risk, such as air traffic control [41] and the military [42]. More recently, SG have been used in the medical field to examine, for example, the impact of task interruptions on the medical evaluation of patients. Results have shown not only that these interruptions lengthen the duration of medical evaluations but also that subsequent decisions are more disorganized and deviate from prescribed standards [42].

The use of SG that reproduce an immersive ED environment and triage tasks allows assessing the impact of distractors on triage accuracy and represents an innovation in the field of triage quality improvement. Against this background, we are planning a pilot study to examine the preliminary effects of distractors on nurse emergency triage accuracy using an SG that we are developing specifically to simulate an ED environment. The study will have three main objectives: (1) develop the SG, (2) assess the feasibility and acceptability of our study design and the SG [43, 44], and (3) evaluate the primary effects of distractors on nurse emergency triage accuracy.

### Theoretical framework

The theoretical framework chosen for the study is an adapted version of the Systems Engineering Initiative for Patient Safety (SEIPS) model [45]. It is a framework frequently used in human factors analysis and patient safety research to gain insight on performance in different healthcare settings, including emergency departments [46–48]. The model comprises three dimensions: work system, processes, and outcomes. It has three main strengths. First, it can describe and operationalize the components of the work environment. Second, it can explain the interactions between the multiple components of the work system and the care processes. Third, it can demonstrate how the interactions between the work system and the care processes impact care outcomes. The model makes explicit the relationship between the work environment and the decision-making process. It distinguishes five components of the work system: person, tasks, tools and technology, environment, and organization.

Specifically, tools and technology refer to the elements that persons use to perform their tasks, such as a computerized care file, an electronic blood pressure monitor, and a telephone. ED studies have shown that technological breakdowns (computer downtime, blood pressure monitor out of order) can cause task interruptions [32, 49]. As this component is commonly explored when new technologies are introduced in the field of care, it will not be studied here, given that the nurses that will participate in the study are proficient in the use of the tools and technologies associated with the triage station.

The person component represents the core variable of the work system and refers not only to all the members of a team, which can include physicians, nurses, and other care providers, but also to the patients themselves and their families. In our study, the focus will be on nurses performing the task of emergency triage. For this component, Carayon et al. [45] recommended examining various elements, including personal, physical, and psychological characteristics, such as knowledge, motivation, and needs [45]. Triage studies have shown, in particular, that nurse education level, triage training, and work experience can influence triage accuracy, as can their aptitude to work fast and their confidence in their decision-making [28, 30, 35, 50].

The tasks component refers to the tasks to be performed by the person. In our study, the task corresponds to triage activities. Carayon et al. [45] proposed exploring elements of this component, such as the variety of tasks to be performed, job demands, and skills required to complete the task. ED studies [32] have identified one key element that disrupts the triage process: task interruptions. A task interruption is “an unexpected temporary or definitive halt to a human activity” [51] (p. 5, free translation). The presence of task interruptions diminishes the operator’s attention by causing it to be redirected elsewhere [32]. This can lead to a loss of information and to decision-making delays and errors [33, 52–54]. Within the framework of our study, the task interruptions that will be incorporated in the clinical vignettes, namely telephone calls, face-to-face communication, and patient requests, were chosen after consulting with the experts of the Swiss Triage Group.

The environment component refers to the physical environment. It is characterized by various elements, including noise, lighting, air quality, and workstation design. Among these elements, ED studies have identified noise as a factor that limits interaction between care provider and patient and as a potential distractor and stressor [31, 34, 55]. It can have a direct impact on productivity and safety in the workplace [56]. Noise is defined as an assortment of sounds perceived as annoying [57]. It is characterized by intensity (decibels), type (continuous, intermittent, variable), duration of exposure (time), and frequency [56]. Research has shown that nurses in emergency clinical units are exposed to continuous ambient noise of more than 65 dB, which exceeds the maximum noise levels recommended by the WHO for hospitals [58–60]. In our study, noise will be considered as a distractor and inserted directly in clinical vignettes. In order to examine the different noises present at an emergency triage station, we will, in conjunction with the experts of the Swiss Triage Group, document these noises through on-site recordings and then select the ones to which triage nurses are most commonly exposed.

The organization component refers to management style and time management, available resources, social relationships, and rules and procedures in place [45]. Nurse triage guidelines mention constraints that bear upon the task, especially limited time [61, 62]. Two studies [49, 63] carried out in European ED have demonstrated the mean duration of nurse triage to be 4 min. In our study, the time required by the nurses to determine the triage score for each clinical vignette will be measured and the variable will be considered in the analyses.

Though the SEIPS model focuses on the work system dimension, it also allows examining the dimensions of process and outcomes. The process dimension informs on the reasoning used by nurses to arrive at clinical decisions [64]. Within the framework of our study, there will be no in-depth examination of the decision-making process per se, only of the final outcome, that is, the SETS® scores (i.e., emergency level) that nurses assign to patients. Finally, the outcomes dimension will be analyzed in terms of accuracy of assigned emergency level. The pilot study will not examine the outcomes dimension in connection with employees and the organization.

### Purpose of the study

This pilot study has a double purpose. First, we wish to evaluate the acceptability and the feasibility of both our study design and the SG we developed to simulate the triage activity in an ED and reproduce an immersive work environment complete with distractors. Second, the study will also assess the preliminary effects of distractors (noise and task interruptions) on the triage accuracy of emergency nurses in the French-speaking part of Switzerland.

### Research questions

The questions asked in connection with the evaluation of preliminary effects are the following:

1. What is the acceptability level of our study design and the SG?
2. What is the feasibility level of our study design and the SG?

The questions asked in connection with the evaluation of preliminary effects are the following:

Primary question:

1. What are the individual and combined effects of distractors on ED nurse triage accuracy?

Secondary questions:

2. How are nurse sociodemographic variables and personal characteristics related to triage accuracy?
3. What is the relationship between nurse-perceived confidence and triage accuracy?

### Proposed hypotheses

We propose the following hypotheses informed by the SEIPS theoretical model [45]: (1) our protocol is easy to implement in a real-life situation and emergency nurses can easily participate and are happy to adhere to our study, (2) exposure to a distractor will lower nurse triage accuracy and inter-rater reliability, (3) exposure to two distractors will lower nurse triage accuracy and inter-rater reliability more so than exposure to only one distractor, (4) nurse work experience is positively related to triage accuracy, and (5) nurse-perceived confidence is positively related to triage accuracy.

## Method

### Study design

In order to evidence the effects of noise and task interruptions on nurse triage accuracy, we will carry out a 2 × 2 factorial randomized controlled trial [65]. A factorial design will be used given that two independent variables will be considered (noise and task interruptions) and that we will evaluate not only the effect of each variable on the dependent variable (nurse triage accuracy), but also their combined effect [65, 66]. The design will follow the CONSORT guidelines [67] and drive the structure of the trial and the choice of control, an analysis of study benefits, the quality and reliability of the intervention, a description of the population, the randomization procedure, and the statistical analysis plan.

This factorial design will allow us to create four groups: one control group and three experimental groups (A, B, C). While triaging the clinical vignettes, nurses in the control group will not be exposed to distractors. Nurses in experimental groups will be exposed to noise (group A), task interruptions (group B), or both noise and task interruptions (group C). Nurses will be block-randomized across the four groups by a computer program. The groups will be of equal size or as similar as possible in this regard.

The study design comprises repeated measures. We will collect sociodemographic and personal data from participants before they begin evaluating the clinical vignettes. Then, during the evaluation of each clinical vignette, the following data will be gathered systematically: (1) emergency level assigned, (2) level of perceived confidence in emergency level assignment, and (3) duration of each clinical vignette evaluation. Upon completing the evaluation of the 20 clinical vignettes, the participants will be asked to complete a questionnaire on the acceptability of the SG.

### Population and sampling

This multi-site study will be carried out in EDs where the SETS® is used. This is the case in 20 private and public care facilities in the five cantons of the French-speaking part of Switzerland (Geneva, Vaud, Fribourg, Jura, Neuchâtel). The population will consist of nurses who perform triage in these facilities. This corresponds to an accessible population of 454 nurses. The eligibility criteria will be similar to those used in previous studies of triage accuracy [27, 35, 68]. Specifically, to participate in our study, nurses must (1) consent to participate and (2) perform triage in one of the EDs where the SETS® is used. Nurses will be block-randomized across the four groups. We established a size of 20 nurses per group, for a total of 80. Around 18% of the accessible population is included, a non-negligible percentage that leaves, nonetheless, an ample margin for the recruitment for the future randomized control trial.

This sample size allows the inclusion of, at least, few nurses from each hospital providing an overview of all the emergency units of the hospitals in French-speaking Switzerland. Therefore, we can assume that our sample provides a sufficient heterogeneity to test acceptability and feasibility of our protocol. Moreover, assuming an accurate triage rate of 0.85 for the control group, a decline of 0.1 in the experimental groups, and an intraclass correlation (that is, between vignettes triaged by a same nurse) of no more than 0.03, we estimated through simulations that each group would need to comprise at least 20 participants to obtain a power of at least 0.80 in order to answer the primary research question. Consequently, we will aim to form a convenience sample of at least 80 nurses, and we will cease recruitment once this target is reached.

### Procedure

The study will follow a two-step procedure. The first step will be to develop the SG. This will be done by a multidisciplinary team comprising IT engineers and designers who will handle the technical elements of the SG and healthcare professionals who will create realistic content, that is, the clinical vignettes, the distractors, and the visual appearance of each on-screen element. The second step will be to recruit participants in the EDs and deliver the SG.

#### Procedure for constructing the serious game and clinical vignettes

First, a pre-design session will be held to define the different steps in the construction of the SG called *SGTRI* and to describe the tasks to be performed by the game developers, the clinical partners, and the research team. Second, the design session will cover all the elements required to recreate the ED triage environment, such as the graphic interface, clinical vignettes, adjunctive distractions, and triage tasks. The SGTRI will be designed on and operated from an open-source platform called *Wegas* (http://www.albasim.ch). To develop *SGTRI* and allow it to evolve, the research team will use a logical graphic interface that may include audio-visual elements adaptable to needs and scenarios. This graphic interface will consist of a virtual 2D waiting room that will be the stage for different animated clips where patients may arrive by ambulance or by foot and other healthcare workers (paramedics, doctor) may be present. A triage workstation will be recreated from 2D plans based on the ED triage observations of a designer on the research team. This triage station will be equipped with all the devices used by nurses under the circumstances (e.g., triage form, clock, computer).

The clinical vignettes will be developed in conjunction with an emergency clinical specialist nurse, an emergency medicine professor (the initiator of SETS® development), and a certified emergency nurse. All these experts have numerous years of experience in the emergency field and with the triage process. A series of 20 interactive clinical vignettes will be created based on a retrospective review of real cases in an emergency department. The clinical vignettes will be constructed following the three quality guidelines proposed by Evans et al. [69]: (1) each vignette must simulate situations faced by participants, which will be the case in this study; (2) each vignette must be different and entail a specific decision to be made, which in our case will be to assign an emergency level; and (3) using well-designed vignettes must produce a highly generalizable “real-life” triage process. For each clinical vignette, the emergency level will be validated by mutual agreement by a group of four experts (two staff physicians and two nursing experts), in strict compliance with the criteria and definitions of the SETS® [20]. This will constitute the gold standard. For our study, the clinical vignettes will involve the medico-surgical issues most encountered in ED and all four emergency levels of the SETS will be covered. To create an immersive ED environment, we will select noises and task interruptions based on real-time observations and recordings by members of the research team in different ED triage settings and on a review of the scientific literature, using the instrument developed by Johnson and colleagues [70] for classifying task interruptions during nurse triage.

In each experimental group (A, B, C), 10 interruptions and/or noises will be introduced in different clinical vignettes. The distribution of task interruptions (type, number, and duration) will follow a predetermined sequence generated by the researchers. The SG will leave the nurse participants the choice of responding or not to some task interruptions (e.g., an incoming telephone call) but will require them to respond to others (e.g., a patient inquiring about the wait time). The noise will correspond to the soundscape (observed values) of triage stations. The research team will modulate the noise exposure condition by varying the form, length of exposure, and intensity of the ambient noise (e.g., conversation, telephone ringtone). The intensity of the noise exposure will range from 35 (A) to 85 dB (A), the maximum level at which no auditory protection is required [71]. To immerse nurses in the created soundscape and eliminate extraneous noise, nurses will be required to wear headphones during the SG session.

Lastly, triage nurse activities will be identified and designed to correspond as much as possible to real-life tasks. These will be the most common tasks performed by nurses at triage, such as taking the patient’s clinical history through a list of predetermined questions (e.g., Are you in pain?), measuring vital signs (e.g., blood pressure), recording patient clinical values, and transcribing the emergency level and chief health complaint. Once the *SGTRI* is designed, a pre-test will be planned with eight clinical emergency experts. The aim of the pre-test will be to assess all the processes to correctly implement the SG during the recruitment phase.

#### Procedure for participant recruitment and SG delivery

First, the aims of the research will be presented at the annual meeting of ED SETS® users. Second, all the care facilities with an emergency unit in the five cantons of the French-speaking part of Switzerland (Geneva, Vaud, Fribourg, Jura, Neuchâtel) will be contacted to validate their interest in participating in the study. Third, nurses interested in participating in the study will receive an information and consent form. The definitive list of participants will be drawn up after consent forms are signed. Nurses will be block-randomized across the four groups just before starting the SG by order of arrival for the test. Each participant will then be assigned an identification number when they start the SG. Fourth, the study data will be collected directly in each ED by the research team. During the session, the research team will be on site to ensure nurse participants are correctly assigned and to ensure fidelity of SG session delivery.

SG delivery will comprise four stages. First, participants will receive a 30-min research project information and training session led by a member of the research team. Second, the participants will run through a training session composed of two clinical vignettes that will not be included in the analyses. During this session, the participants will familiarize themselves with the equipment (headphones, laptop) provided by the members of the research team. Third, once the training session is completed, each nurse will be able to start their SG session when ready. The nurses will have 2 h to complete the 20 clinical vignettes. This corresponds to the average number of patients triaged at an ED over 2 h. To establish a controlled set for the SG, participants will be isolated in a meeting room previously prepared by the head nurses in each participating emergency unit. Members of the research team will be on site to provide technical support, if needed, and to document any technical problem that might occur during the SG. Fourth, once the 2 h has elapsed, each participant will stop the game session even if not all 20 vignettes have been evaluated. All the data collected during the SG session will be automatically recorded, and they will be saved on a secured server located in Switzerland. After their 2-h SG session, the participants will return to their workplace.

### Instruments

Sociodemographic data, both personal (gender, age, family situation) and professional (employment status, total number of years of experience, number of years in current department), will be collected through a questionnaire developed on the basis of elements gathered in previous studies of triage accuracy [27, 35, 72].

The clinical decision-making of the nurse participants will be judged on the emergency level that they assign based on the SETS® criteria. Following their clinical reasoning, nurses assign patients an emergency level from 1 to 4. The scale has been the focus of various independent studies [20, 73] where computerized clinical vignettes were used with ED nurses and paramedics.

Nurse level of confidence in their clinical decision-making will be measured using a visual analogue scale from 0 to 100 [74]. This scale will be presented to nurses after each emergency level assignment. The question asked will be: “Now that you have completed this clinical vignette, how confident are you of the emergency level that you have assigned?” Nurses will rate their confidence from 0 to 100, with 0 corresponding to “I am not at all confident of my decision” and 100 to “I am fully confident of my decision.” Visual analogue scales allow measuring the intensity of a subjective experience and are widely used in clinical settings [66]. In a study where the scale was used by 69 nurses in a triage situation, the researchers reported no problems with its utilization [35].

The feasibility of the SG will be assessed on the basis of criteria drawn from Sidani and Braden [44] and Feeley et al. [75], including accessibility of target population, appropriateness of inclusion and exclusion criteria, participation rate, withdrawal rate after starting SG, presence and frequency of problems during delivery of SG (understanding, utilization, clarity), presence and frequency of missing data and outliers, and participant satisfaction with SG.

The acceptability of the SG will be measured using a French version of the self-administered AttrakDiff 2 inventory [76] initially developed in German by Hassenzahl and colleagues [77]. This 28-item scale allows evaluating the hedonic and pragmatic qualities of interactive systems such as SG. Each item takes the form of a 7-point scale (− 3 to + 3) on which to rate a quality expressed by semantic differentials, that is, a pair of antonyms. It comprises four subscales: usability, functionality, social impact, and attractiveness. For each item, the respondent must choose between seven answers book-ended by the semantic differentials. A mean score and standard deviation are calculated for each dimension, taking account of certain inverted items [76]. The values between 0 and 1 are considered neutral. Dimensions are deemed positive if scored between + 1 and + 3 and negative if scored between 0 and − 3, in which case the SG needs to be improved. The psychometric properties (validity and reliability) of the French-language scale are entirely satisfactory, having obtained a Cronbach’s α of 0.75 for each of the dimensions [78]. A supplementary question in the form of a visual analogue scale from 0 to 100 will be added to examine how realistic the nurses perceive the SG to be relative to their professional activity.

### Data analysis plan

The nurse participants (expected *N* = 80) will be the analysis units for the descriptive analyses, and the assigned triage scale scores will be the analysis units for the correlational analyses and some descriptive analyses (number of nurses multiplied by number of vignettes—expected *N*: 80 × 20 = 1600). The following data analysis plan will be carried out to answer the research questions: First, the collected data will be verified (compliance with inclusion criteria, identification of missing data and outliers). Second, the data on the nurses (sociodemographic and professional) will be analyzed via descriptive statistics, both univariate (mean, median, standard deviation, interquartile range, and absolute and relative frequency) and bivariate (contingency table and marginal frequency). Third, triage accuracy will be measured by the level of agreement between the answers given by the nurses and the gold standard established by the experts. For each nurse, the scores assigned to each clinical vignette will be compared against the gold standard. The results of the comparison will be a three-level multinomial variable: accurate triage (nurse score same as gold standard), overtriage (score higher than gold standard), and undertriage (score lower than gold standard). Over- and undertriage frequencies will be used to describe the triage accuracy of the four groups, that is, the control group and the three experimental groups: noise (A), task interruptions (B), and noise and task interruptions (C). Fourth, to examine the individual and combined effects of the distractors on the triage accuracy of the nurse participants, the groups will be compared against one another using a random-intercept multinomial regression model. For all the analyses, the statistical significance level will be *p* ≤ 0.05. All the data will be analyzed using the R statistical software [79].

### Ethical considerations

Each nurse from the emergency units selected for the study will receive a written information letter explaining how the study will be conducted, what their participation entails, and what data protection measures will be taken. Each nurse will then be able to take all the time they need to decide whether to participate in the study, without the decision having any consequence whatsoever for their career. The research team will have access only to anonymous data. All data will be deleted after data analysis. To participate in the study, the nurses will have to sign a consent form, which will be stored in accordance with the recommendations of the Swiss Human Research Ethics Board (Canton of Vaud, Switzerland). The time that the nurses spend evaluating the clinical vignettes with the SG will count as work hours.

## Discussion and conclusion

Triage is considered a dynamic and complex nursing activity that requires a high level of concentration in a work environment marked by frequent task interruptions, an unpredictable workload, and a noisy physical layout [80]. These three elements may influence the accuracy of the emergency levels that nurses assign to patients [32, 52]. Our research project will seek to test the effects of distractors on triage accuracy through the use of an SG that simulates the ED environment. The project will overcome many limitations of previous studies by accounting for a distractive environment and providing a safe setting for patient triage assessment. The results of our study will inform the design of a follow-up large-scale study that will not only unscramble the impact of distractors on the triage process but also identify sociodemographic factors that may play a role in triage accuracy. This could provide emergency units with critical information that will allow them to adapt the work environment and allocate health workers more efficiently and effectively.

Today’s technological advances have made it possible to create tools such as SG to optimize the learning and evaluation processes in many different fields. The most salient success stories so far have perhaps been in medicine and aviation. SG, such as the one that we are developing and studying, are tools that hold tremendous promise not only to help us better understand triage accuracy and the impact of distractors but also to help us benchmark the triage process and improve its quality. They may also be used in education and ED training as a complement to written scenarios and in-person simulations.

In experimental investigations, SG allow collecting a wide array of precise measurements, maintaining full control of simulated events, modulating parameters with rigor, measuring performances in detail, and controlling trigger events better [78]. SG, as presented here, have considerable advantages for simulation and empirical research. All their benefits, however, must rely on a strong methodological design [81]. Based on the analysis of 12 studies that evaluated the effects of task interruptions on health activities, Sanderson and Grundgeiger concluded that three aspects were decisive in creating a reliable simulation: (a) fidelity, that is, “the apparent realism of the investigative context in relation to the domain itself”; (b) control, that is, “the measures taken to ensure that the conclusions of an investigation are specific and logically defensible”; and (c) potential generalizability, that is, “the potential for depth of insight and breadth of application of conclusions” [81] (p. 87). These aspects will be carefully controlled in developing our SG and in any further investigation of it.

Our research project has various limitations, some linked to the general use of SG and others specific to SGTRI. The general elements have to do essentially with the use of human-machine interaction to simulate human-human interaction. For example, the current status of SG technology requires that some forms of interaction, such as verbal exchanges, be reduced or even eliminated completely in favor of other forms of interaction, such as written exchanges. Moreover, SG limit sensorial interactions to sight and hearing, whereas the real triage process involves all of the senses. As for the limitations specific to SGTRI, our SG presents a series of static situations that do not evolve over time and reduces the complexity of triage by removing elements, such as patient flow management and the re-evaluation of complex cases. Furthermore, distractors and environmental configurations can vary widely across ED, and *SGTRI* might not be representative of the layout of triage desks. These limitations are the consequences of both technical limitations and deliberate choices to focus our SG on a few but well defined and structured elements: patient evaluations and the influence of external distractors on the triage process.

*SGTRI* also presents a number of strengths. First, it offers a flexible instrument that can be easily tailored to numerous research projects, whether on the effects of distractors or other subjects, such as the effects of tiredness or the impact of nurse sociodemographic and professional characteristics on triage. Second, in education, it offers an efficient means of easily and quickly updating the knowledge of triage nurses. Unlike other training approaches, SG can provide a very large number of nurses with individualized and flexible training that can be adapted to their needs and requirements by, for example, modulating or controlling the level of difficulty or the amount of time to spend on each training session. Although the use of technology-enhanced simulation to train ED healthcare professionals is an innovative solution, authors underscore the importance of applying a rigorous methodology and outcome measures when using technology in the simulation field [82]. Numerous studies that have evaluated the effectiveness of SG using different outcome measures (e.g., clinical decision-making and cognitive and perceptual effects) [83–85] have found them to be reliable tools when robust methodologies, designs, and outcome measures are used. In this context, the results of our pilot study will provide useful information to test and improve our SG, our research hypotheses, and our methodology.

## Data Availability

The data sets generated and/or analyzed in the course of this study will not be publicly available owing to privacy regulations. However, they will be made available in anonymized form by the corresponding author upon reasonable request.

## Abbreviations

CV: Clinical vignette
ED: Emergency department
SETS®: Swiss Emergency Triage Scale
SEIPS: Systems Engineering Initiative for Patient Safety
SG: Serious games
